# Advanced Nanocellular Foams: Perspectives on the Current Knowledge and Challenges

**DOI:** 10.3390/nano11030621

**Published:** 2021-03-02

**Authors:** Daniel Cuadra-Rodriguez, Suset Barroso-Solares, Javier Pinto

**Affiliations:** 1 Cellular Materials Laboratory (CellMat), Condensed Matter Physics Department, University of Valladolid, 47011 Valladolid, Spain; dcuadra@fmc.uva.es; 2 BioEcoUVA Research Institute on Bioeconomy, Cellular Materials Laboratory (CellMat), Condensed Matter Physics Department, University of Valladolid, 47011 Valladolid, Spain; sbarroso@fmc.uva.es

Nanocellular polymers (i.e., cellular polymers with cells and walls in the nanometric range) were first produced in the early 2000s, with the works of Yokoyama et al. [1] being the main precedents in this field, producing nanocellular structures by using supercritical carbon dioxide. However, it was not until a decade later that this research field started to grow significantly, attracting several international research groups in the quest to obtain cellular polymers with cells in the nanocellular range [2]. From 2010 to 2014, the basis of bulk nanocellular foam production was established, and the CO_2_ gas dissolution foaming technique rapidly proved to be the most suitable production route for such materials (details and theoretical basis of this technique can be found elsewhere) [2,3,4]. Continuous technical advances (e.g., higher saturation pressures, lower saturation temperatures, faster pressure drop rates) and diverse nucleating agents, from inorganic nanoparticles to block copolymers, provided a broad collection of cellular polymers with submicrometric and nanometric cells [2]. Although quite diverse polymers allowed the achievement of submicrometric cells, amorphous polymers such as polyetherimide (PEI), polystyrene (PS), and, notably, poly (methyl methacrylate) (PMMA) provided the best nanocellular structures, with cell sizes even below 100 nm and significant density reductions [2].

The continuous progress achieved in terms of the cell size and relative density decrease obtained during these years raised the expectations regarding these advanced materials, aiming to reach even smaller cell sizes and larger porosities. Moreover, from theoretical predictions and the previous experience of the positive effect of the cell size decrease to the micrometric range on the physical properties of these materials, the nanocellular polymer foams were expected to present exceptional physical properties. For instance, nanocellular foams could have improved thermal insulation, low dielectric constant, enhanced mechanical performance, or even optical transparency [2,3]. This exceptional development of the field and these expectations were carefully analyzed by Costeux [2] in 2014, pointing out other open challenges, such as the production of open-cell nanocellular structures, the development of strategies to remove or avoid the formation of the solid outer skins typical of gas dissolution foaming, and the need to develop continuous processes to produce such materials.

Accordingly, as the nanocellular foams field has continued to grow since 2015, it is necessary to perform a critical analysis of its progress, evaluating the fulfilment of the created expectations, the advances in its understanding, the resolved and ongoing challenges, and the key focus points through which to maintain the growth of this field. Herein, this analysis is structured as follows. First, the latest advances in the production of nanocellular foams are briefly summarized, highlighting the most relevant achievements and challenges. Then, the progress in the study of their physical properties is discussed, followed by an evaluation of the success in overcoming the challenges mentioned above. Finally, the main ideas extracted from this critical analysis are presented, identifying the most challenging, and promising, current focus points in this field, as well as potential applications identified in the last few years, which is the main aim of this Special Issue.

Decreasing the cell size is, without doubt, the motivation behind research on nanocellular foams. In the last few years, several works have achieved the production of nanocellular foams with cell sizes below 100 nm. On the one hand, some crystalline polymers have finally provided such features, but showing severe limitations in terms of density reduction, as cell nucleation and growth can only occur following the pattern of the amorphous phase between crystalline lamellae [5]. Some of the best results achieved, by optimizing the polymer formulations and foaming parameters, allowed the achievement of high-density poly(lactic acid)-based and polypropylene-based nanocellular foams, respectively, with average cell sizes of around 30 and 55 nm and cell densities (measured on the amorphous foamed regions) of around 10^15^ cells/cm^3^ [6,7]. Nevertheless, there are very few perspectives on obtaining even medium-density nanocellular foams, as the identified key parameters to obtain nanometric cells are the increase in crystallinity and the number of crystalline spherulites, in order to act both as nucleating agent and growth boundaries for the cells (i.e., limiting the expansion of the foam) [6,8,9].

On the other hand, several amorphous polymers have shown the capability of producing nanocellular foams (e.g., poly(ether-block-amide), acrylonitrile–butadiene–styrene terpolymers, poly(aryl ether ketone), and thermoplastic polyurethane) [10,11,12,13,14,15], but just a few achieve cell sizes below 100 nm (e.g., polyphenylsulfone (PPSU), polysulfone (PSU), and PMMA) [16,17,18,19]. The main interest in exploring the use of PPSU and PSU is that they offer a relatively high service temperature, not achievable with PMMA. Bernardo et al. [16] proved that nanocellular PPSU foams could be obtained in a wide range of saturation pressures (15–30 MPa, saturation temperature 25 °C) and foaming temperatures (150–220 °C), reaching cell sizes of around 20–30 nm, cell nucleation densities over 10^15^ nuclei/cm^3^, and relative densities between 0.65 and 0.75. Meanwhile, Guo et al. [17] achieved similar results employing PSU, 5 MPa of saturation pressure, saturation temperatures below 0 °C, and foaming temperatures between 70 and 150 °C. Nevertheless, PMMA retained the lead as the most promising and versatile polymer in developing nanocellular polymer foams. Both homogeneous and heterogeneous nucleation routes continue to be explored with this aim.

The increase of the CO_2_ solubility on the PMMA, generally by increasing the saturation pressure or decreasing the saturation temperature, as well as the optimization of other foaming parameters (e.g., depressurization rate, foaming temperature, and time) and the polymer matrix chemistry are the available tools for researchers working on the homogeneous nucleation route. Ono et al. [18] employed ultra-high saturation pressures (50–100 MPa), saturation temperatures between 0 and 20 °C, ultra-fast depressurization (>1.25 GPa/s), and foaming temperatures between 30 and 110 °C to produce nanocellular PMMA foams with cell sizes below 50 nm, cell nucleation densities over 10^15^ nuclei/cm^3^, and relative densities down to 0.44. Yeh et al. [19] studied the effect of the molecular weight on the foaming behavior of PMMA. They found that high molecular weights (M_w_ = 81 kg/mol) are more suitable to decrease the cell size, although they present more difficulties for density reduction. In particular, using a saturation temperature of 0 °C and pressure of 13.8 MPa (CO_2_ solubility of around 38 wt.%) and foaming temperatures of around 80 °C, they reached cell sizes of around 37 nm, cell densities of around 10^16^ cells/cm^3^, and relative densities of 0.25. The same conclusions were achieved by Martin-de-Leon et al. [20] with a saturation pressure and temperature, respectively, of 20 MPa and −32 °C (CO_2_ solubility up to 45 wt.%), and foaming temperatures up to 80 °C to obtain cell sizes of around 75 nm, cell nucleation densities of 10^15^ nuclei/cm^3^, and relative density of 0.24 for the low-viscosity PMMA grade employed (M_w_ = 77 kg/mol, MFI = 8.20 g/10 min), as well as cell sizes around 14 nm, cell nucleation densities of 3.5 × 10^16^ nuclei/cm^3^, and relative density of 0.4 for the high-viscosity PMMA grade (M_w_ = 83 kg/mol, MFI = 1.92 g/10 min).

On the contrary, the heterogeneous nucleation route offers additional tools to the researchers, related to the nature, size, distribution, and physical properties of the additional phases introduced, their proper distribution being the main challenge of this route. Only a couple of works have been able to achieve cell sizes below 100 nm following this approach. Yeh et al. [19] introduced 1 wt.% of SiO_2_ nanoparticles (average size 15 nm) on a PMMA with high molecular weight (M_w_ = 81 kg/mol) and foamed the samples at a saturation temperature of 0 °C, saturation pressure of 13.8 MPa, and foaming temperatures of around 80 °C, achieving inhomogeneous cellular structures with nanocellular regions with cell sizes of around 70 nm and cell densities around 3.5 × 10^14^ cells/cm^3^. It should be noted that, in this case, the introduction of the nanoparticles negatively influenced the cellular structure, as better results achieved by homogeneous nucleation under the same foaming conditions were previously mentioned. Shi et al. [21] produced blends of PMMA with poly(vinylidene fluoride) (PVDF), finding that a self-assembled structure of PVDF microcrystals induced by the CO_2_ appears on the samples during the foaming process. These well-dispersed microcrystals were proven to act as nucleating agents, achieving cell sizes of 87 nm and relative densities of 0.86 by foaming PMMA/PVDF blends with 40 wt.% of PVDF, with the small cell size and high relative density being related to the rigidity of the semicrystalline phase. Although the results obtained by the heterogeneous nucleation route in the last few years have not been quite successful in achieving cell sizes below 100 nm, a few additional works are worth mentioning due to their contribution to the understanding and optimization of this approach. Liu et al. [22] proved that the modification of silica nanoparticles with poly(dimethylsiloxane), a polymer with low surface energy and high CO_2_-philicity, enhances the cell nucleation efficiency of the nanoparticles from 0.04 to a remarkable value of 0.5. Moreover, they found that modified nanoparticles with sizes of around 80 nm present higher efficiencies than nanoparticles with sizes of around 40 nm and explain this behavior in terms of the line tension effects on the nucleation process. Interestingly, they propose that the same theoretical approach could be applied to justify the nucleation efficiency near 1 previously found in PMMA/MAM blends [23]. Needle-like sepiolite nanoparticles were also introduced in PMMA by Bernardo et al. [24], finding that their orientation during the extrusion of the precursor materials induces significant anisotropy (up to 2.15) in nanocellular foams. This anisotropy was explained in terms of the cell coalescence around the needle-like sepiolite, which could act as spot for cell wall rupture. Finally, the foaming of PMMA/MAM blends has been extensively studied, paying particular attention to the use of very low MAM amounts (0.1 wt.%), which were shown to be capable of inducing nanocellular structures similar to those obtained by MAM amounts from 1 to 10 wt.%, while providing a relative density reduction up to 50% [25]. In particular, very low MAM amounts are dispersed along with the PMMA matrix without generating micelles. In this way, these PMMA/MAM samples retain the nucleating effect of the soft blocks of the MAM copolymer, while avoiding the spherical geometries of the micelles, which are known to limit the expansion of the cells and therefore the density reduction [4].

The promising results briefly summarized above clearly show that the objective of cell sizes below 100 nm and low relative densities has still not been achieved. The gas dissolution foaming approach still has potential to induce higher CO_2_ solubilities, or even to improve the followed process [26], while further work on very low amounts of block copolymer could enhance the obtained results. Among these approaches, the use of block copolymers (or other nucleating agents) presents the advantage of requiring less demanding foaming conditions (a further increase in CO_2_ solubility using higher pressures or lower temperatures involves increasing production costs).

However, simple geometrical considerations and the physical dimensions of the polymer chains are enough to demonstrate that cell sizes equal to or below 10 nm and low densities cannot be achieved with close-cell geometries [27]. On the contrary, Estravis et al. [27] proposed that open-cell geometries could offer this possibility, reaching cell sizes below 20 nm and porosities over 0.9 simultaneously if cell strut thicknesses of 1.5 nm are assumed. Although the assumed cell strut thickness could be extremely low (struts or walls below 10 nm have still not been reported, while thicknesses over 20 nm are the most common) [25,28], open-cell nanocellular structures should be further studied aiming to achieve low-density nanocellular foams. Nevertheless, among the highlighted works, none of them obtained an open-cell structure able to reach very low densities. Yeh et al. [19] and Guo et al. [17], respectively, obtained nanocellular connected structures or bicontinuous nanocellular structures in the cell walls due to spinodal decomposition, but not highly-porous open-cell structures such as those obtained in an early work of Pinto et al. [29].

The expectations of achieving enhanced physical properties by the production of nanocellular polymers were the main motivation behind the development of these materials. In the last few years, significant knowledge about the mechanical, optical, and thermal properties of these materials has been developed. Wang et al. [9,30] have proposed that the nanocellular structure obtained in PMMA/TPU and PP/PTFE foams induced remarkable improvements in the impact strength, impact toughness, and tensile toughness regarding microcellular PMMA or PP foams, providing an interesting and detailed discussion of the mechanisms potentially involved. However, the complexity of the studied systems, in which both the nanostructured TPU and the nanofibrillated PTFE present a notable influence on the mechanical properties, makes it difficult to analyze the effect of the nanocellular structure independently. On the contrary, Martin-de-Leon et al. [31] developed a specific study on the influence of the cell size on the compression and three-point bending mechanical tests of PMMA foams, comparing nanocellular foams with cell sizes from 20 to 84 nm and microcellular foams with cell sizes of around 1 µm (all of them with comparable densities). No dependence of the relative Young’s modulus and yield strength upon cell size was found, while the relative fracture toughness was found to increase when the average cell size decreased from the micro to the nanocellular range.

The potential optical transparency of nanocellular foams obtained from transparent amorphous polymers was a controversial topic for some time [3]. However, recently, strong experimental evidence has shown that optical transparency can be achieved when the cell sizes of nanocellular PMMA foams are below 1/10 of the visible wavelengths due to the strong size effect on the Rayleigh scattering [32]. These results encourage further work on this topic, as transparent nanocellular polymers could be extraordinary materials to improve the thermal insulation of windows, therefore having a global impact in decreasing energy consumption.

The thermal conductivity of nanocellular foams is one of its most promising physical properties, as the presence of the Knudsen effect, experimentally validated in nanocellular polymers in 2015 [33], could lead to the production of high-efficiency thermal insulators. However, the failure to obtain nanocellular foams with both cell sizes well below 100 nm and low relative densities has hindered till now the achievement of such insulators. Only one nanocellular PMMA/TPU foam, with a cell size of around 205 nm and relative density of 0.125, has been reported showing thermal conductivity of 24.8 mW/mK (below that of air, 26 mW/mK) [30], although the same authors have recently theoretically estimated that such low thermal conductivity cannot be reached with the features of the reported foam [34]. Recent experimental evidence on the interaction of infrared (IR) radiation with nanocellular foams proved that the thermal conductivity due to the radiation term has a major effect on these materials (i.e., nanocellular foams are almost transparent for IR radiation) [35]. Taking this into account, the minimum thermal conductivity achievable for PMMA foams has been estimated to be around 32 mW/mK, reached with very low densities and cell sizes of around 150 µm [34]. Accordingly, the potential super-insulator behavior of nanocellular foams, taking advantage of the Knudsen effect, only could be achieved if additional mechanisms to abate the radiation term are incorporated [34,35]. An alternative approach to taking advantage of the Knudsen effect while avoiding the limitations of the density reduction and the undesired transparency to IR radiation was proposed by Bernardo et al. [36]. In this work, a model to predict the thermal conductivity of foams presenting a bimodal cell size distribution (with nanometric and micrometric populations) was developed and validated with experimental results. The model was shown to provide accurate predictions for such materials, as well as for monomodal cell structures with broad cell size distribution. Moreover, it showed potential for the thermal conductivity reduction of bimodal cellular structures with low-density regions with cells in the microcellular range (decreasing the conduction through the solid phase and avoiding a significant contribution of the radiation) and medium-density regions with cells in the nanocellular range (decreasing the conduction through the gaseous phase). Therefore, further developments in the pursuit of super-insulator nanocellular foams should focus on strategies to decrease the radiation term without affecting the nanocellular structure [37], as well as continuing to seek to decrease the overall density of the foam.

Another thermal property which suffers changes in nanocellular polymer foams is the glass transition temperature (T_g_) of the amorphous polymer matrix. The increase in the T_g_ has been widely reported on PMMA nanocellular foams [19,38] and seems to be related to the molecular confinement of the polymer chains on the nanometric cell walls and struts [28]. However, the remarkable implications of these effects at the molecular level require further research. In addition to experimental approaches, significant advances could be achieved in this field with the application of simulation theoretical approaches at the molecular level, such as molecular dynamics (MD). Recent works have shown the potential of these techniques in the study of nanocellular polymers. Estravis et al. [27] employed MD to analyze the stability of gas–polymer structures in the nanocellular range, finding that the behavior of the internal pressure in the polymer is a key factor in the minimum stable cell size achievable. Moreover, Zhang et al. [39] studied the behavior of ultrathin PMMA films following the same approach, investigating the failure of cell walls during cell growth on nanocellular foams.

A couple of the challenges identified by Costeux in 2014 are still to be discussed. On the one hand, no significant advances have been achieved on the removal or avoidance of the formation of the solid skins in the outer layers of nanocellular foams obtained by CO_2_ gas dissolution foaming [4]. It should be noted that these solid outer layers could hinder the expansion of the foams, being also a limiting factor that limits the application of nanocellular foams in fields such as sensing, catalysis, and filtration (fields which would require mostly open-cell nanocellular foams to take actual advantage of their very high surface area). On the other hand, scarce progress has been achieved in the continuous or semi-continuous production of nanocellular foams. Costeux and Foether [40] presented the first successful results in the fabrication of nanocellular foams by a complex extrusion foaming system in 2015, obtaining PMMA-based foams with cell sizes of 300 nm and porosities of around 70%, but no further results in this area have been reported since. Meanwhile, Wang et al. [9] reported for the first time the fabrication of PP/PTFE nanocellular foams following an injection molding foaming approach.

Accordingly, several current main challenges in the advanced nanocellular polymer foams field have been identified. First, the simultaneous reduction of the cell size and density requires further technical developments (to increase the CO_2_ solubility) exploring the use of dispersed non-nanostructured block copolymers, but mostly focusing on the development of open-cell nanocellular foams. Second, there is still room for the understanding and optimization of the physical properties of these materials. New approaches to decrease the radiation term on the thermal conductivity of nanocellular foams are urgently required, while the potential of bimodal cellular structures should be further explored. Moreover, the production of transparent nanocellular foams requires additional progress to identify a compromise between their transparency and their thermal insulation. In addition, as a key aspect in improving the understanding of these materials and their physical properties, additional work is required in the study of the increased T_g_ and confinement effect of nanocellular polymers, using both experimental and theoretical approaches employing MD or similar techniques. Third, the development of strategies to effectively remove the solid outer skins, or, even better, to avoid their formation, as well as continuous processes capable of producing nanocellular foams, are essential for several potential applications and the future industrialization of these materials.

Additionally, new applications should be considered and tested for these materials. For instance, PMMA-based materials have shown the capability of treating polluted water [41,42,43], an application which could benefit from the very high surface area of nanocellular open-cell foams. PMMA has also been employed as a support for nanoparticles with photocatalytic activity as well as to develop contact lenses, the transparency of nanocellular PMMA foams being a potential advantage in these applications [42,43,44,45]. Finally, nanocellular polymer films have been proposed as water- and oil-repellent anti-reflection coatings, this particular application being quite suitable for transparent nanocellular foams [46].

## Data Availability

Data sharing not applicable.
